# Parallel- and serial-contact electrochemical metallization of monolayer nanopatterns: A versatile synthetic tool en route to bottom-up assembly of electric nanocircuits

**DOI:** 10.3762/bjnano.3.14

**Published:** 2012-02-16

**Authors:** Jonathan Berson, Assaf Zeira, Rivka Maoz, Jacob Sagiv

**Affiliations:** 1 Department of Materials and Interfaces, The Weizmann Institute of Science, Rehovot 76100, Israel

**Keywords:** AFM (SFM), bipolar electrochemistry, electrochemical metal deposition, monolayer patterning, nanolithography, self-assembled organosilane monolayers

## Abstract

Contact electrochemical transfer of silver from a metal-film stamp (parallel process) or a metal-coated scanning probe (serial process) is demonstrated to allow site-selective metallization of monolayer template patterns of any desired shape and size created by constructive nanolithography. The precise nanoscale control of metal delivery to predefined surface sites, achieved as a result of the selective affinity of the monolayer template for electrochemically generated metal ions, provides a versatile synthetic tool en route to the bottom-up assembly of electric nanocircuits. These findings offer direct experimental support to the view that, in electrochemical metal deposition, charge is carried across the electrode–solution interface by ion migration to the electrode rather than by electron transfer to hydrated ions in solution.

## Introduction

The quest for a chemical methodology applicable to the bottom-up fabrication of planned electric nanocircuits that can be effectively addressed from the external macroscopic world continues to pose major synthetic challenges. Metal growth or deposition on or within a preformed template structure has been successfully used in the fabrication of various metallic nanoscale objects and periodic nanostructures [1–12]; however, a comprehensive chemical methodology applicable to the planned assembly of metallic nanostructures of arbitrary shape and size, spanning variable length scales, is yet to be advanced.

Our laboratory has devoted ongoing efforts to an approach centered on the use of patterned organic monolayers as stable templates on top of which guided self-assembly of other selected materials of interest, organic as well as inorganic, can take place [13–28]. To this end, a monolayer-patterning methodology, referred to as constructive lithography (CL), has been advanced, which allows nondestructive local electrooxidation of the top –CH_3_ groups of a self-assembled OTS/Si monolayer (highly ordered monolayer assembled on silicon from *n*-octadecyltrichlorosilane precursor molecules [22,29]) to –COOH functions [14,16]. The hydrophobic and chemically inert OTS surface is thus locally converted to a hydrophilic and chemically active one. Patterns of such OTSeo (electrooxidized OTS) regions surrounded by the unmodified OTS monolayer (denoted as OTSeo@OTS/Si) were produced using either conductive SFM (scanning force microscope) probes that can serially inscribe OTSeo features on lateral length scales from nanometers to tens of micrometers (constructive nanolithography, CNL) [14–15,18,27] or conductive stamps, suitable for one-step parallel printing of OTSeo features extending over much larger surface areas, typically beyond the micrometer (constructive microlithography, CML) [16,22].

Recently, we demonstrated a two-step CL patterning and pattern metallization process, referred to as *contact electrochemical patterning and transfer* (CEP–CET), whereby OTSeo features are first printed or inscribed on a target OTS monolayer by using a stamp electrode consisting of a patterned silver film on OTS (Ag/OTS@OTS/Si) or a silver-coated SFM tip electrode, and then in-situ metallized by direct electrochemical transfer of the metal from the patterning electrode itself [30]. CEP–CET is implemented in an unconventional "contact electrochemical" configuration, similar to that employed in previously studied constructive-lithography patterning processes [14,16,18,22]. In this configuration, the patterning electrode (metal-film stamp or metal-coated scanning probe) touches the target monolayer through an interfacial water layer of molecular-to-nanoscale thickness (adsorbed on the metal grains by capillary condensation from a humid, ambient atmosphere), which fulfils the role of the electrolyte. To achieve local electrooxidation of the target monolayer (CEP step), the target is biased positively (anode) with respect to the patterning electrode, whereas for metal transfer (CET step), the polarity of the applied bias voltage is reversed so that the stamp or the SFM probe now acts as the anode and the target monolayer as the cathode [30].

Metal-on-monolayer features resulting from the serial CEP–CET process executed with a moving SFM tip were shown to correspond to the OTSeo features defined in the pattern inscription step (CEP), whereas those produced with a stamp (parallel mode) were replicas of the stamp metal features [30]. Since patterned metal-film stamps could be easily fabricated by metal evaporation through transmission electron microscopy grids used as contact masks, the parallel CEP–CET process has hitherto only been implemented on lateral length scales larger than several micrometers. Here we report proof-of-concept experimental results demonstrating the feasibility of a different and more versatile contact electrochemical strategy for the nanoscale fabrication of diverse metal/monolayer patterns, based on the finding that metal deposition by the CET process is possible only on monolayer surfaces exposing metal-ion-binding functions (e.g., –COOH, –S–S–, –SH) [31].

## Results and Discussion

**Figure 1:**
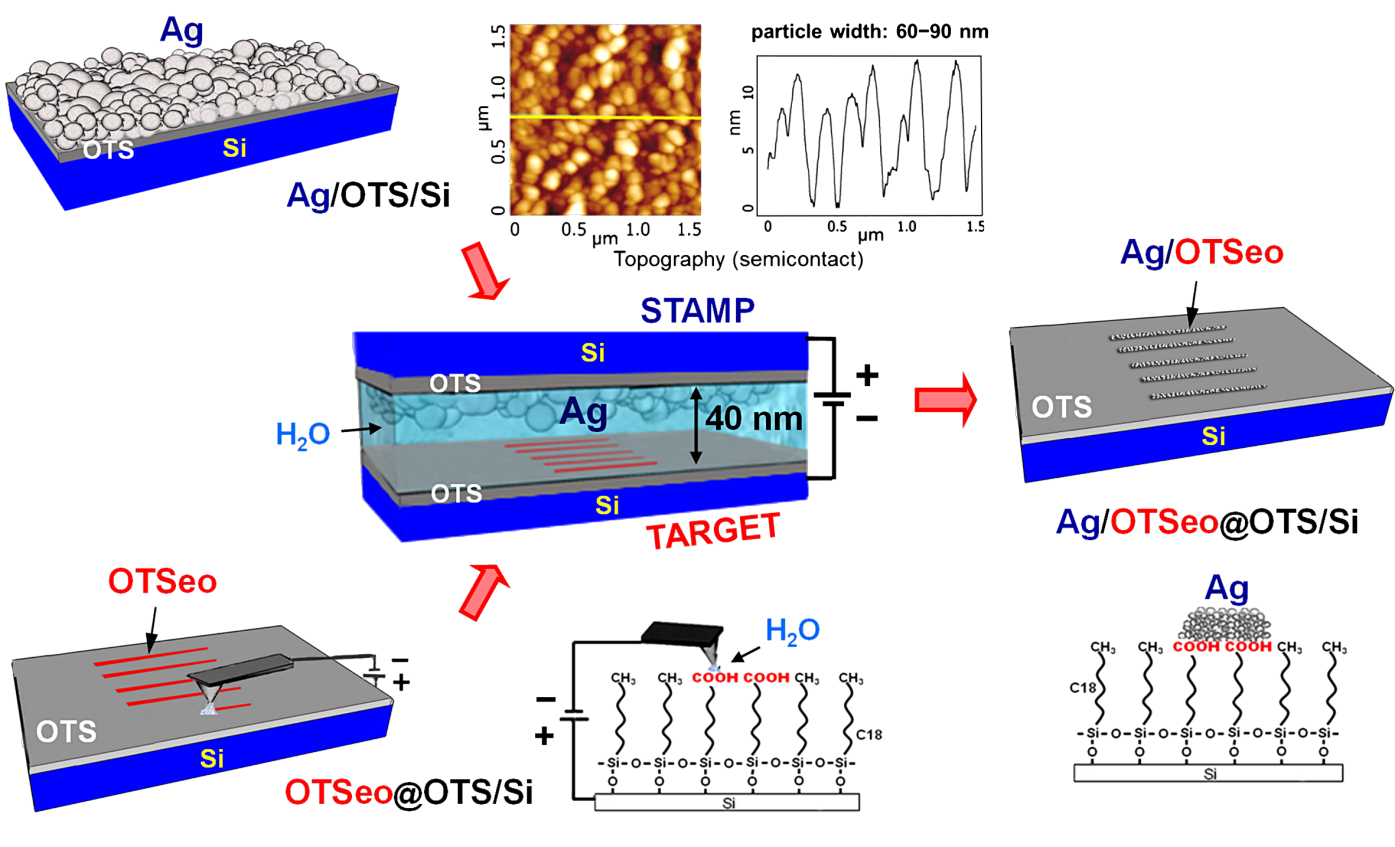
Scheme of parallel-contact electrochemical metallization of a OTSeo@OTS/Si template nanopattern (target, cathode) consisting of an array of parallel OTSeo lines serially inscribed with a conducting SFM tip on a self-assembled OTS monolayer on silicon (CNL process, bottom left). Selective silver deposition on the OTSeo lines of the patterned monolayer (right) is achieved by contact electrochemical transfer of the metal (center) from a stamp (anode) consisting of a thin (~40 nm), granular Ag film deposited by metal evaporation on the entire surface (2–4 cm^2^) of an OTS/Si monolayer specimen (top) [30]. The granular morphology of such silver-film stamps is evident in the displayed SFM image. During the application of the bias voltage (center), the stamp–target sandwich is equilibrated with a water-saturated atmosphere (see Experimental section).

**Figure 2:**
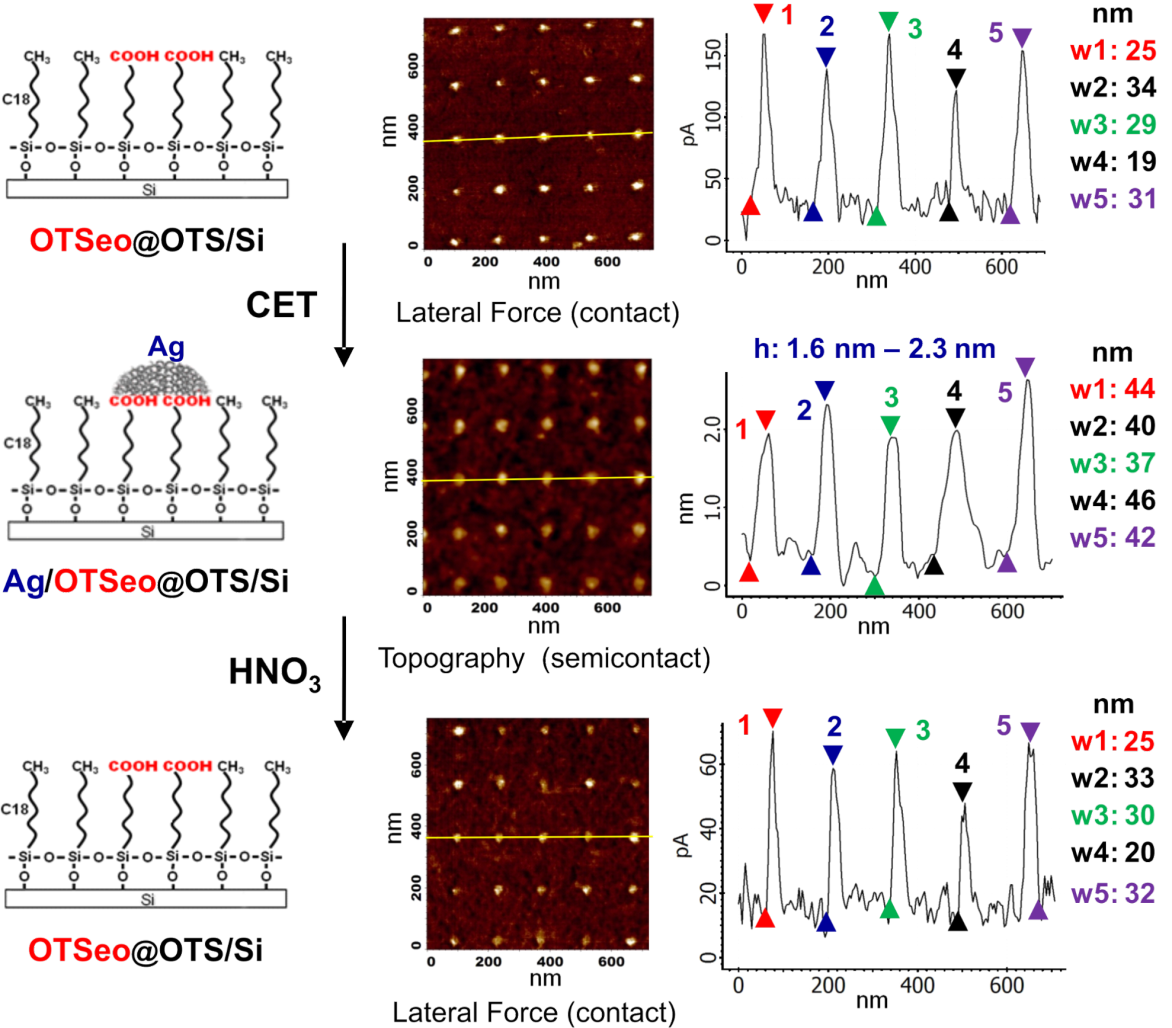
SFM images (and distance–height profiles along the marked lines) acquired after each step during the fabrication of an array of silver/monolayer nanodots by the contact electrochemical process depicted in Figure 1, as well as after removal of the metal by dissolution in nitric acid (see Experimental section): (Top row) the initial target array of monolayer-template nanodots (OTSeo@OTS/Si); (middle row) array of metal/monolayer template nanodots (Ag/OTSeo@OTS/Si); (bottom row) recovered array of OTSeo@OTS/Si monolayer-template nanodots, after removal of the deposited metal. Contact-mode topographic images of the metal-free dots (top and bottom rows) show a similar scan-dependent weak contrast relative to the OTS background, indicative of the structural integrity of the OTSeo monolayer template (see [30] and Supporting Information File 1, Figures S2 and S3).

**Figure 3:**
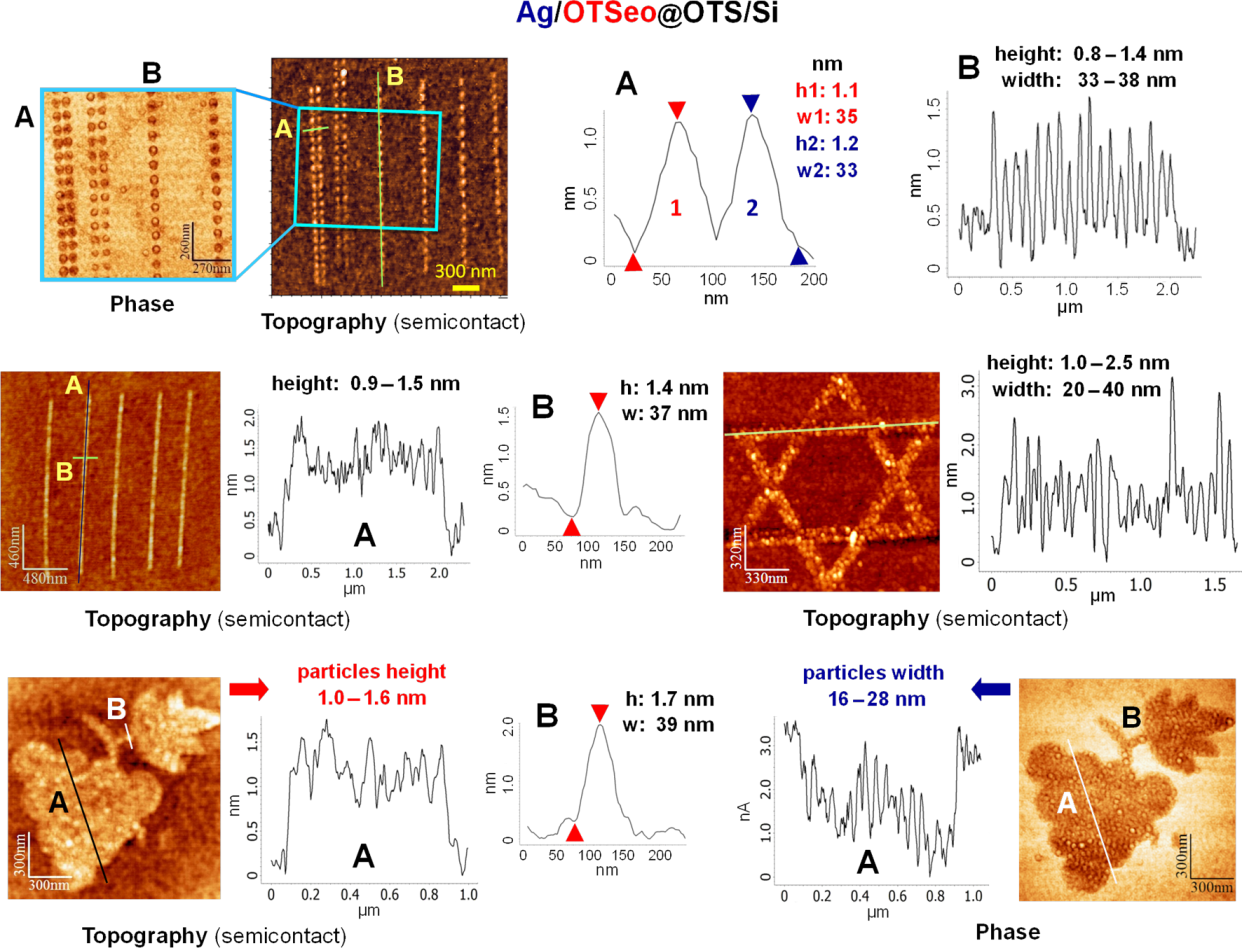
Semicontact SFM images (and distance–height profiles along the marked lines) of different silver/monolayer nanostructures fabricated in the same manner as the nanodots in Figure 2 (see text and Experimental section).

**Figure 4:**
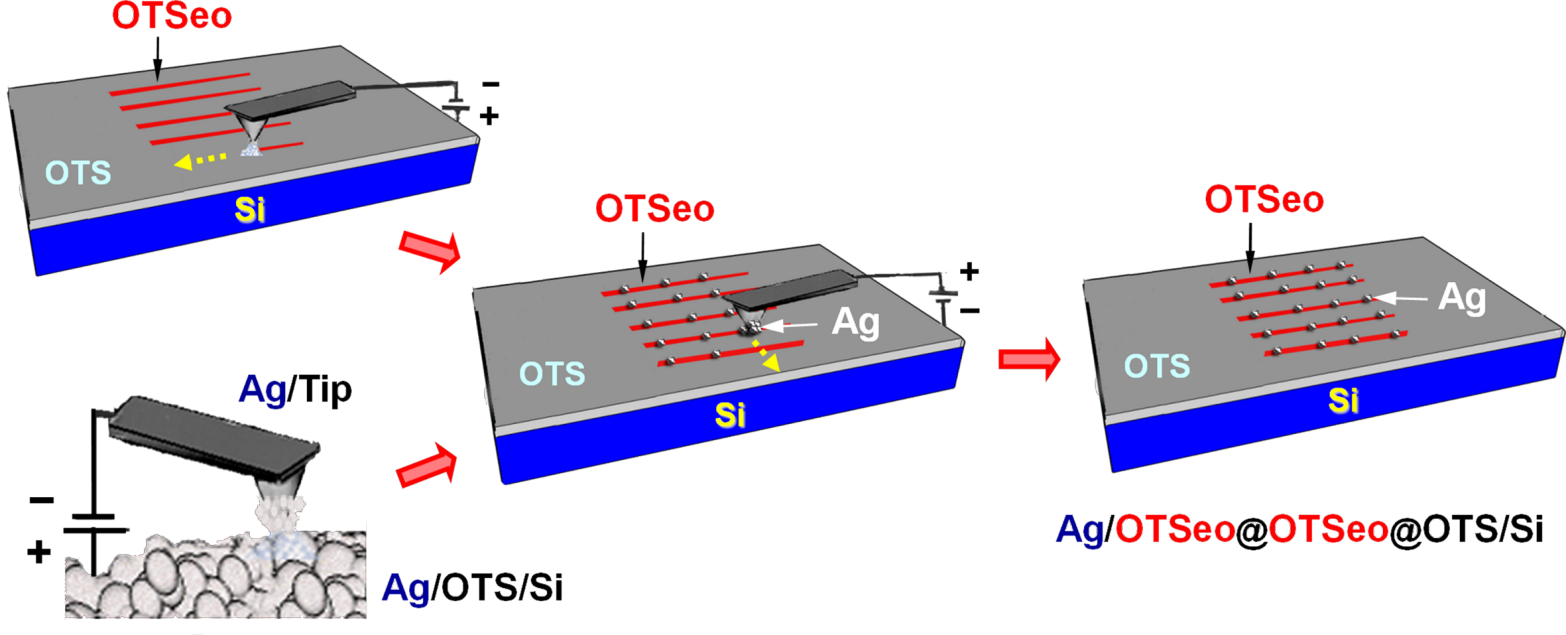
Scheme of serial-contact electrochemical metallization of selected sites within the OTSeo lines of a OTSeo@OTS/Si template nanopattern: (top left) inscription of OTSeo lines with a conductive SFM tip (CNL process); (bottom left) loading of silver on a conductive SFM tip by contact electrochemical transfer from a thin silver film evaporated on a OTS/Si monolayer; (center) selective-contact electrochemical transfer (CET) of silver from the silver-coated tip to selected sites along the OTSeo lines, implemented by moving the positively biased tip (mobile anode) across the OTSeo lines that play the role of cathode for metal deposition (see text); (right) resulting pattern-within-pattern array of silver/monolayer nanodots (Ag/OTSeo@OTSeo@OTS/Si denotes Ag/OTSeo sites within metal-free OTSeo regions located within the unmodified OTS/Si monolayer). As in the parallel CET process (Figure 1), no metal is transferred from tip to surface in a dry atmosphere and in the absence of an appropriate voltage bias (see Supporting Information File 1, Figure S1).

**Figure 5:**
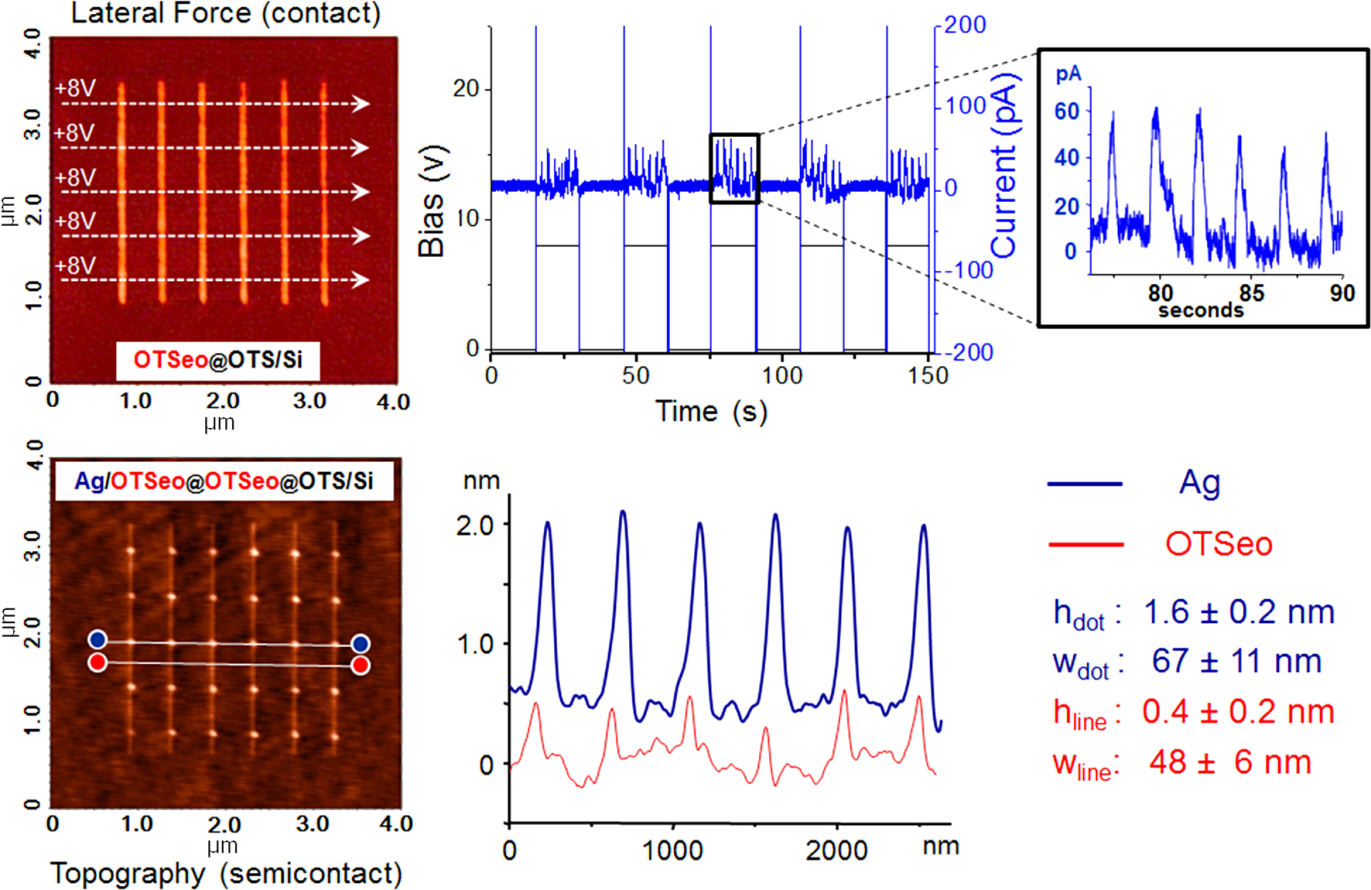
Fabrication of a rectangular array of 30 silver/monolayer nanodots by the serial CET process outlined in Figure 4 (see Experimental section): (Top row, left) five horizontal tip excursions across the array of six parallel OTSeo lines used in the assembly of the Ag/OTSeo nanodots (indicated by white arrows in the lateral force SFM image of the OTSeo lines); (top row, right) plots of tip bias voltage (+8 V, black curve) and corresponding current (blue curve) versus time recorded during each tip excursion (tip moving in contact with the surface at a constant speed of 250 nm/s); (bottom row) topographic semicontact-mode SFM image of the resulting dots@lines pattern (Ag/OTSeo@OTSeo@OTS/Si) and distance–height profiles along the middle row of Ag/OTSeo dots (blue curve, shifted vertically for clarity) and a closely located row of silver-free OTSeo crossing points (red curve). The average heights and widths listed on the right refer to all 30 dots and OTSeo crossing points. Contact-mode topographic images of this dots@lines pattern and a comparative analysis of the contact- and semicontact-mode topographic images (revealing the artifactual nature of the former) are provided in the Supporting Information File 1 (Figures S2 and S3).

As shown below in Figure 1, Figure 2 and Figure 3, all OTSeo features of a serially inscribed OTSeo@OTS/Si nanopattern can be simultaneously metallized in a parallel CET operation performed with an unpatterned, thin silver-film stamp (Ag/OTS/Si), whereas precise delivery of metal to selected surface sites within selected OTSeo features of such a monolayer nanopattern can be realized in a serial mode, by moving a positively biased silver-coated SFM tip along a planned trajectory across the patterned area of the monolayer (see below in Figure 4 and Figure 5).

According to Figure 1, upon the application of a voltage bias between stamp and target, with stamp positive and target negative, silver is selectively transferred to the OTSeo lines of the target monolayer only, thus producing a pattern of metallized OTSeo lines surrounded by the unmodified OTS monolayer. As discussed in the following, the selectivity of silver deposition on the OTSeo lines follows from the fact that Ag^+^ ions generated electrochemically at the metal stamp (anode) are transported through the adsorbed water film, acting as an electrolyte, to the target monolayer (cathode), where effective nucleation and growth of stable metal grains (following the reduction of Ag^+^ ions to neutral atoms) can occur only at those surface sites that bind the ions, which correspond to the carboxylic acid terminated OTSeo lines of the template nanopattern. Examples of metal/monolayer nanopatterns fabricated by this parallel metallization process are given in Figure 2 and Figure 3. It should be emphasized that no metal is transferred in a dry atmosphere and in the absence of a bias voltage applied between stamp and target as shown in Figure 1, regardless of the mechanical force pressing the two surfaces together and the time of contact.

Metal-free and metal-covered OTSeo sites such as those displayed in Figure 2, Figure 3, and Figure 5 (see below) can be unambiguously identified in lateral-force (contact-mode) and semicontact-mode (tapping) topographic images, respectively [30]. This is a consequence of the large difference in the polarity of the outer exposed functions of OTSeo (–COOH) and OTS (–CH_3_), which gives rise to a corresponding large difference in the frictional force exerted on a tip moving in contact with a patterned monolayer surface of this kind. For the same reason, the corresponding contact-mode topographic images yield false height contrast, dependent on the direction of tip motion relative to the sample (see Supporting Information File 1, Figures S2 and S3). This is a characteristic feature of monolayer patterning by constructive lithography, which generates highly heterogeneous hydrophilic–hydrophobic monolayer surfaces [14,16,18,22,30]. Correct height values of the deposited silver, as displayed in Figure 2, Figure 3, and Figure 5 (see below), were thus obtained from semicontact-mode images.

An examination of the different patterns displayed in Figure 2 and Figure 3 indicates that OTSeo template features with local widths (*w*) below ca. 30 nm guide the formation of thin, platelike silver particles that span the entire width of the template and tend to grow beyond its boundaries while maintaining heights (*h*) on the order of 1–2 nm. On small dotlike template sites, this metal growth mode yields discrete Ag nanodots (Figure 2 and Figure 3, top row), whereas continuous Ag nanowires with a bamboolike structure of higher and lower metal features are formed on narrow template lines (Figure 3, middle row left). Identical deposition conditions applied to wider template features result in multiple nanoparticles with similar heights and somewhat smaller average lateral dimensions (Figure 3, middle row right and bottom row). Regions A and B in the bottom-row images in Figure 3 are representative of metal growth on both the wide regions and the narrow lines of the same template feature, respectively. Because of the high density of nanoparticles in region A, the lateral resolution of individual particles in the topographic image of this region (left) is poor, particles widths being here obtained from the simultaneously recorded phase image (right).

The size and lateral organization of metal particles formed on the different OTSeo template patterns in Figure 2 and Figure 3 are seen to differ not only from those characteristic of the granular silver film used as the stamp (see Figure 1), but also from one another. This is rather remarkable, given the fact that all these patterns are located on same target specimen and their electrochemical metallization was simultaneously performed with the same silver-film stamp. Equally remarkable is also the fact that no metal was deposited within the unmodified portions of the OTS surface and that undamaged template patterns (OTSeo@OTS/Si) could be regenerated by dissolving the electrochemically deposited metal (Figure 2). It was further observed that more metal is deposited with longer electrochemical stamping times under the same applied voltage bias, and metallic features were seen growing also laterally in a mushroomlike fashion (beyond the area of the underlying OTSeo template), without affecting the integrity of the surrounding OTS surface. Carrying out contact electrochemical experiments as in Figure 1 with target monolayers patterned by mask-defined local photocleavage of the OTS alkyl tails [22,27], it was finally established that no metal is deposited in bare regions present within an OTS monolayer.

In line with previously reported findings [30], these observations unequivocally demonstrate that: (i) The CET mechanism of metal transfer from stamp to target is electrochemical rather than adhesion-promoted [32–35], involving dissolution of stamp-metal grains (anode), ionic transport through an ultrathin water film adsorbed on the metal grains, and subsequent nucleation and growth of new metal grains at the target monolayer (cathode); (ii) metal grains can nucleate and grow only on monolayer-template surfaces exposing chemically active functions that bind the respective metal ions, the morphology and lateral distribution of the resulting metal features thus depending on the local dimensions and topology of the template features on which the metal grains nucleate and grow; (iii) there is no metal nucleation and growth in pinhole defects in the organic monolayer that might not be detected by the SFM imaging, so that metal deposited by the present CET process necessarily resides only on the outer surface of the monolayer template, with full preservation of its structural integrity. Recent electrical measurements indeed confirm the absence of metal–silicon conductive paths in Ag/monolayer/Si structures fabricated in this manner. The CET process thus yields metal-on-monolayer deposits with no contacts to the underlying solid substrate, in-principle different from those usually produced in conventional electrochemical deposition on thiol/gold monolayers [36–45], which may occur in the monolayer-free regions of a destructively patterned monolayer [36–42], underneath the monolayer [41–42], or on top of the monolayer with metallic contacts reaching the metal substrate through defect sites in the monolayer [39–45].

In view of these experimental observations, it was anticipated that by replacing the metal stamp with a positively biased metal-loaded SFM tip that can be programmed to move across the surface according to a predefined trajectory, it should be possible to create more complex "pattern-within-pattern" structures by serial delivery of metal to selected surface sites within selected OTSeo template regions of a prepatterned OTS monolayer. For example, in the case of OTSeo lines (Figure 4), since metal is not deposited on the pristine OTS monolayer, metal transfer from tip to surface should be confined to the intersection regions of each cathodic OTSeo line with the directions of motion of the anodic tip. Experimental results confirming the feasibility of this approach are given in Figure 5 and Figure S1 (Supporting Information File 1).

In Figure 5, each on–off switching of the bias voltage, at the beginning and end of a horizontal tip excursion, respectively, is seen to be accompanied by a pair of sharp, capacitance-related current spikes of opposite sign, whereas smaller and broader positive current spikes, on the order of 30–50 pA, clearly correlate with tip-to-surface metal transfer within each tip/OTSeo crossing region. The total transferred charge (deduced from the integrated area of each current spike) is, however, significantly larger than that corresponding to the amount of deposited metal, which indicates that other bias-dependent processes, competing with the electrochemical metal transfer from tip to surface, also contribute to the total measured current [30] (see proposed model in the following). As is further evident in Figure 5, the platelike silver nanodots fabricated by this serial CET process are similar to those produced in the parallel CET mode (Figure 2 and Figure 3); however, the serial process offers the option of precise control over the generation of discrete nanoparticles at isolated sites within each OTSeo template line, in contrast to the uncontrollable fusion of adjacent nanoparticles on the narrow OTSeo lines or their random lateral distribution on the wider OTSeo regions in the parallel process (Figure 3).

**Figure 6:**
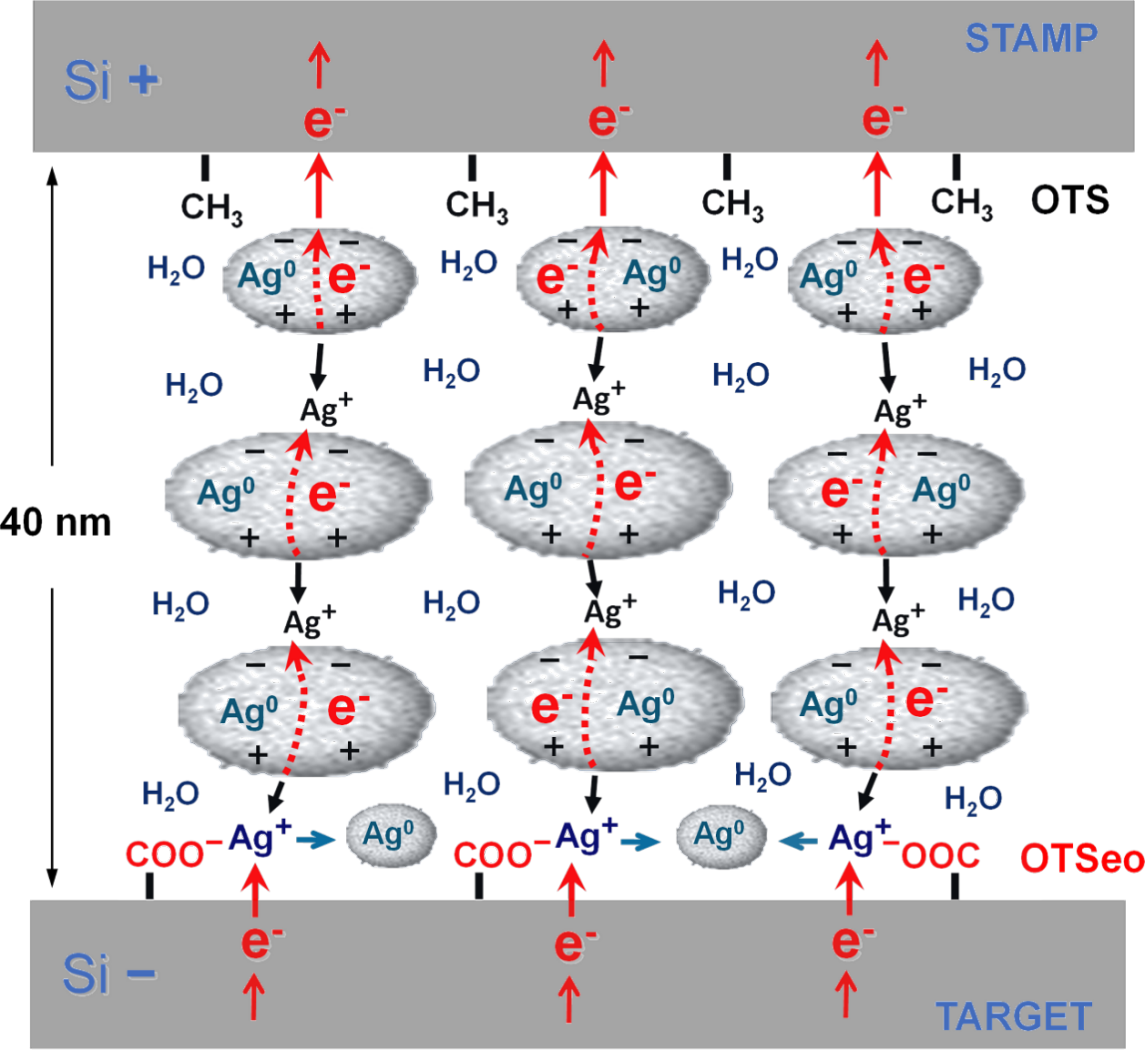
Proposed bipolar electrochemical mechanism of metal transfer from a thin, granular silver-film stamp (Ag/OTS/Si) to a carboxylic acid terminated target monolayer (OTSeo/Si) in a contact electrochemical configuration like that depicted in Figure 1 (see text). Key features emphasized in this schematic illustration (not to scale) are the nanoscale thickness of the granular silver film, the molecular–nanoscale thickness of the water film (electrolyte) adsorbed on the silver grains by capillary condensation from a humid atmosphere, the gradual dissolution of silver grains next to the OTS monolayer (stamp), and the nucleation of new silver grains at the OTSeo monolayer (target) upon the reduction of Ag^+^ ions chemisorbed on the OTSeo surface as –COO^−^Ag^+^ species [30]. Note that several closely located Ag^+^ ions need to be simultaneously discharged at the target monolayer in order to generate a stable metal cluster.

The origin of the remarkable surface selectivity of metal deposition in these CET processes may be understood with reference to the schematic electrochemical model depicted in Figure 6, which highlights some of the salient features of the metal transfer and its high surface selectivity. As shown before [30], an ultrathin layer of water adsorbed on the metal grains of a granular Ag film stamp exposed to a humid atmosphere may convert each such grain into a tiny bipolar electrode [46–48], from which Ag^+^ ions are released at its anodic side (+, facing the negative electrode) and redeposited as elemental silver at its cathodic side (−, facing the positive electrode). Since no metal ions are supplied to the cathodic side of the topmost grains in the metal film, these grains will gradually dissolve and eventually disappear. Concomitantly with their dissolution, metal is deposited on the surface of the OTSeo target monolayer through the reduction of chemisorbed Ag^+^ ions (by electrons supplied by the negative silicon electrode) followed by the nucleation and growth of new metal grains. These metal grains grow at the expense of the dissolving stamp grains next to the positive electrode, thus resulting in gradual transfer of metal to the target monolayer. As emphasized before [30], in addition to the ionic current responsible for the metal transfer, the total measured current is expected to include also contributions from competing Faradaic processes, such as the electrolysis of water, as well as from direct electronic current through closely spaced metal grains in the thin silver film. The experimental data in Figure 5 support this view.

The crux of the selective electrochemical deposition of silver on the OTSeo surface has to do with the fact that single Ag^0^ atoms are highly reactive and therefore short-lived [49–51]. Reaching a critical nucleus size that would allow further stable growth of a larger metal grain [52] is, thus, not possible unless a critical number of silver atoms are simultaneously generated through the reduction of an equal number of closely located silver ions. This can be accomplished at a target surface covered by a silver-binding monolayer such as OTSeo, in which the dense –COOH functionality of the organic monolayer facilitates the establishment of a sufficiently high local concentration of chemisorbed Ag^+^ ions through the conversion of carboxylic acid groups to the carboxylate salt (–COO^−^Ag^+^). In contrast with OTSeo, metal deposition by this mechanism on a pristine OTS surface is not possible because of the very low probability of nucleation and growth of metal grains on such a surface devoid of ion-binding functions [53]. Since the local concentration of hydrated silver ions in solution in front of an OTS monolayer should be much lower than that of Ag^+^ ions chemisorbed on the OTSeo surface, while their distance from the silicon substrate is considerably larger, isolated silver atoms that might eventually be generated as a result of the reduction of such ionic species by electrons reaching the solution through the OTS monolayer are expected to rapidly return to their ionic state (by electron transfer to surrounding water molecules [54–55]) or redeposit on preexisting stamp-metal grains, before aggregation into stable metal clusters residing on the OTS surface can occur.

## Conclusion

The high selectivity achieved in the contact electrochemical deposition of silver on monolayer-template features exposing metal-ion-binding functions created by constructive nanolithography offers a versatile and reliable synthetic tool for the deliberate assembly of various metal-on-monolayer nanostructures, to be used as building blocks in the bottom-up fabrication of entire nanocircuits [56]. This is possible, as the present electrochemical methodology is compatible with low-conductivity substrates [30] and the deposited metal features reside on an extremely robust insulating layer of variable thickness (here the organic silane monolayer plus the native silicon oxide underneath it) that separates them from the substrate and provides effective electrical insulation over a range of useful applied voltages lower than those applied during the monolayer patterning and metallization processes themselves. While rapid formation of multiple circuit elements, such as arrays of metal nanodots and nanowires, may be achieved by using metal-film stamps in the parallel-metallization mode (Figure 2 and Figure 3), serial generation of metal/monolayer nanoobjects occupying only a limited portion of the total area of the respective monolayer-template features (such as the nanodots in Figure 5) should permit more complex structures to be realized through consecutive template-guided assembly steps [14,17–18,24,27]. For example, in this manner one could easily fabricate various collinear sequences of metal and semiconductor [14,17] nanodots and nanowires, confined to any desired layout of monolayer-template lines, straight, curved, parallel or intersecting. The precise deposition of metal at selected locations on the selected template lines is guaranteed here by the inherent electrochemical selectivity of the CET process, which precludes metal deposition on the unpatterned OTS surface.

For the application of this methodology to the fabrication of an entire addressable nanocircuit, the present nanoscale metallization processes need to be combined with analogous CET processes applicable on much larger length scales [30], which would enable the assembly of micro- and macroscale metal/monolayer contact electrodes. Work toward the realization of such circuits and their electrical–structural characterization is currently in progress.

As far as basic electrochemical aspects are concerned, it is of interest to note that the present findings offer direct experimental support to the recent arguments raised against the usually adopted model of electron transfer from the electrode to a metal ion in solution as the mechanism of charge transfer across the electrode–solution interface in electrochemical metal deposition [54–55]. Indeed, the exclusive deposition of silver on the Ag^+^ binding (OTSeo) sites of nondestructively patterned OTS/Si monolayers demonstrates that metal ions have to shed their hydration shell and reach the electrode surface before being discharged, rather than being first reduced to neutral atoms by electron transfer to hydrated ionic species in solution [54–55].

## Experimental

OTS/Si monolayer samples and Ag/OTS/Si metal film stamps were prepared following experimental procedures detailed in [22] and [30], respectively. The parallel-contact electrochemical metallization experiments (Figure 1, Figure 2 and Figure 3) were performed as described in [30], using a specially designed electrical stamping device that allows control of the bias voltage, the force pressing the stamp and the target together, and the ambient humidity. In the present experiments, a voltage bias of 3.0 V was applied for 2 min between the silver/monolayer stamp and the target monolayer while the two specimens are pressed together with a force of about 100 N in a water-saturated atmosphere (RH 100%). Deposited silver dots were removed (Figure 2) by immersion in HNO_3_/H_2_O (20% v/v) for ~3 h followed by rinsing with pure water.

All monolayer nanopatterning (CNL) and serial metallization (CET) operations were carried out in the contact mode (in a regime of minimal repulsive force), under controlled humidity at 55–65% RH. A SOLVER P47 SFM system (NT-MDT) was used in the fabrication of the OTSeo@OTS nanopatterns in Figure 2 and Figure 3. The patterns were written with doped-silicon contact probes (CSC-38/AlBS, MikroMasch) or metal-coated contact probes (CSC-37/Ti-Pt, MikroMasch) to which a negative bias of 7.0–8.0 V relative to the surface was applied. Contact-mode images (Figure 2) were acquired with the same probes without an applied electrical bias, and semicontact-mode (tapping) images (Figure 2 and Figure 3) with Silicon AC160TS semicontact probes (Olympus).

The serial CET experiments (Figure 4 and Figure 5) were performed on an NTEGRA Aura SFM system (NT-MDT) specially designed for electrical patterning and structural-electrical characterization of surface architectures [30]. W_2_C-coated HSC20 contact probes (Team Nanotec) were used in the inscription of the OTSeo lines, (under conditions similar to those mentioned above in relation to Figure 2 and Figure 3), whereas the metal-transfer operations were executed with CSC-37/Ti-Pt contact probes (MikroMasch) on which silver was loaded by scanning the surface of an evaporated silver film on OTS for ~5 min with a tip bias of −10 V relative to the silver film. Experimental conditions for the metal delivery from tip to the OTSeo lines (Figure 5) were selected following trial experiments carried out with different applied voltages and tip speeds (Figure S1, Supporting Information File 1). Contact-mode SFM images (Figure 5 and Figures S1, S2 and S3, Supporting Information File 1) were acquired with the patterning tip without an applied bias, and semicontact-mode images (Figure 5) with Silicon AC160TS semicontact probes (Olympus).

## Supporting Information

File 1 Serial CET trial experiments and comparison of imaging results obtained under different SFM imaging conditions.
