# Supplementary material for: Parallel- and serial-contact electrochemical metallization of monolayer nanopatterns: A versatile synthetic tool en route to bottom-up assembly of electric nanocircuits
Source: Beilstein J Nanotechnol. 2012 Feb 16;3:134–43. doi: 10.3762/bjnano.3.14 (PMC3304318; doi:10.3762/bjnano.3.14)
Supplement: File 1 — Serial CET trial experiments and comparison of imaging results obtained under different SFM imaging conditions. [file Beilstein_J_Nanotechnol-03-134-s001.pdf]

Supporting Information

for

Parallel- and serial-contact electrochemical metallization of monolayer nanopatterns: A versatile synthetic tool en route to bottom-up assembly of electric nanocircuits

Jonathan Berson‡, Assaf Zeira‡, Rivka Maoz* and Jacob Sagiv*

Address: Department of Materials and Interfaces, The Weizmann Institute of Science, Rehovot 76100, Israel

Email: Jacob Sagiv* - jacob.sagiv@weizmann.ac.il; Rivka Maoz* - rivka.maoz@weizmann.ac.il

*Corresponding author ‡Equal contributors

Serial CET trial experiments and comparison of imaging results obtained under different SFM imaging conditions

Serial CET – Trial Experiments

CET experiments performed with variable bias voltage applied to the tip and variable tip speed enabled us to select experimental conditions for successful metal transfer to prepatterned OTSeo lines (according to Figure 4, main manuscript). An example of such a trial CET experiment, in which silver was transferred from a silver-coated SFM tip moving across an array of parallel OTSeo@OTS lines like those of Figure 5 (main manuscript) is given in Figure S1. Under the imaging conditions used here (direction of tip motion in the horizontal scan from left to right), the OTSeo lines are visible only in the lateral-force image (a discussion of results obtained under different SFM imaging conditions follows below), whereas the silver dots deposited on the OTSeo lines appear in both the topographic and lateral-force images. Visible deposition of silver is seen to start here at an applied voltage bias of 6 V, which, however, results in dot heights corresponding to only 1–2 atomic layers of silver (below ca. 0.6 nm) that are barely distinguishable in the topographic image. Metal dots with heights in the range 0.5–1.1 nm, produced at a bias voltage of 7 V, are clearly visible in both images (see height–width profile recorded for the top row of dots).

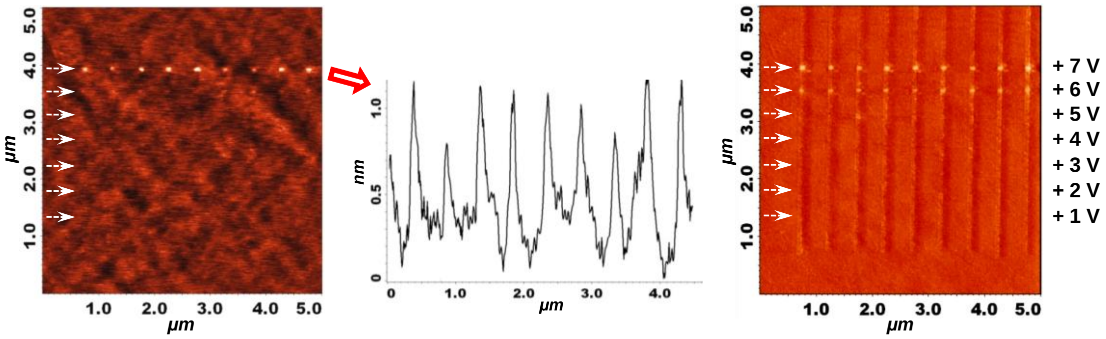

Topography (contact) Lateral Force

Figure S1: Contact-mode SFM images of a silver/monolayer pattern produced in a serial CET experiment (as in Figure 4, main manuscript) in which a different bias voltage was applied to the tip during each of its seven excursions (indicated by the white arrows) across nine vertical OTSeo lines. The tip was moved at a constant speed of 1,000 nm/s while the bias voltage was raised as indicated on the right, from +1 V to +7 V. A distance–height profile is shown along the top row of Ag/OTSeo dots (generated at +7 V).

Comparison of Different SFM Imaging Conditions

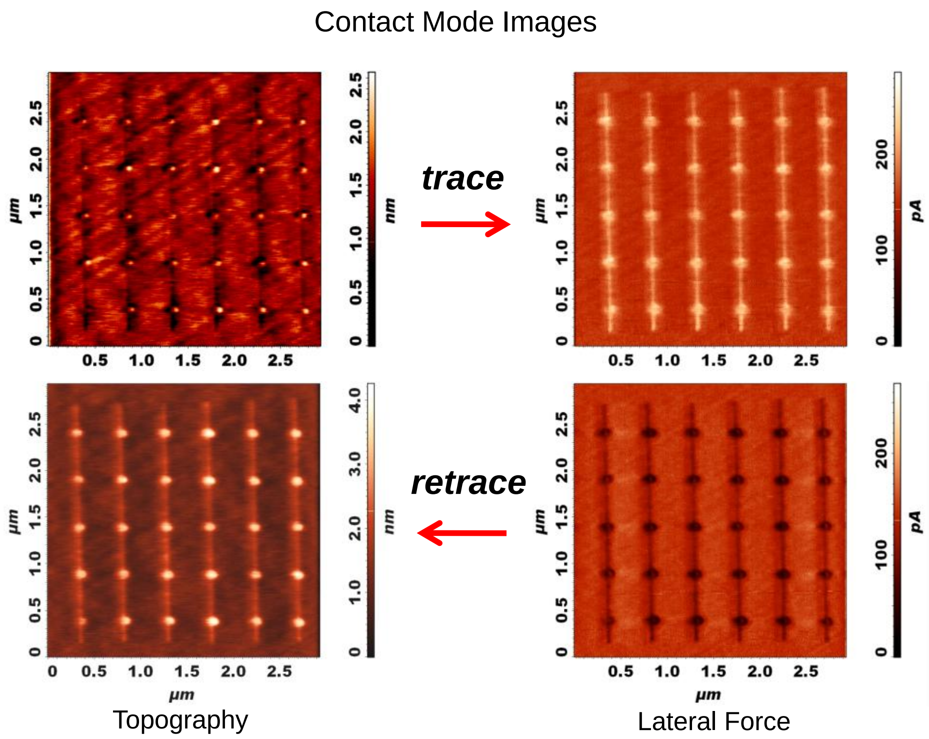

Figure S2: Contact-mode SFM images of the same dots@lines pattern as in Figure 5 (main manuscript). The top-row images (***trace***) were recorded with tip motion relative to sample in the horizontal scan from left to right and the bottom-row images (***retrace***) when the direction of tip motion was reversed.

While the semicontact-mode SFM imaging of Ag/OTSeo@OTSeo@OTS/Si patterns fabricated by the present approach yields identical images regardless of the direction of tip motion relative to the sample in the horizontal scan, rather different images were recorded in the contact mode for the same patterns upon reversal of the direction of tip motion (e.g., Figure S2). Moreover, each of these different topographic images also differs significantly from the corresponding semicontact-topographic image (compare Figure S2 and Figure 5, main manuscript). As discussed before [1-4], the inverted contrast in the ***trace*** and ***retrace*** lateral force images (Figure S2) reflects the higher friction force exerted on the tip when it moves over the hydrophilic OTSeo lines as compared with the hydrophobic OTS background, which causes a torsion of the cantilever in a direction that changes sign when the direction of tip motion relative to sample is reversed. Depending on imaging conditions, the cantilever torsion may give rise to a larger or smaller artifactual topographic effect that manifests itself as an apparent vertical deflection of the tip that changes sign as a function of the direction of tip motion relative to the sample as well and adds to its real vertical deflection

[3,4]. Consequently, as the real height difference between OTSeo and OTS should be very small [5], the OTSeo lines in Figure S2 appear as either depressions below the OTS surface (***trace*** image) or protrusions above it (***retrace*** image), which is a clear manifestation of this imaging artifact [3,4]. As Figure S2 further shows, the same imaging artifact affects the topography of the metal dots as well, detracting from or adding to their real heights as a function of the direction of tip motion. Thus, the apparent dot heights in the ***trace*** and ***retrace*** images in Figure S2 are lower and higher, respectively, than the corresponding dot heights in the semicontact image (Figure 5, main manuscript), which would suggest that intermediate values obtained as the average of ***trace*** and ***retrace*** values may be expected to better approximate the real dot heights.

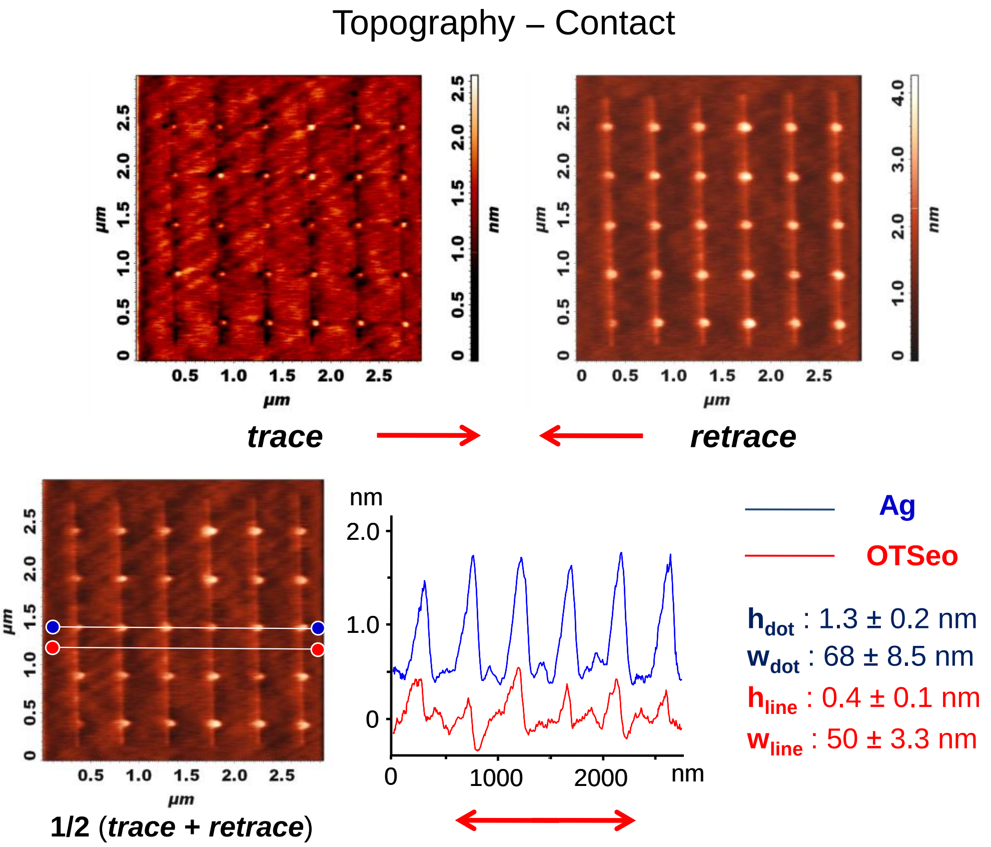

Figure S3: Top row: The same ***trace*** and ***retrace*** topographic images as in Figure S2. Bottom row: Calculated (***trace*** + ***retace***)/2 average image, and distance–height profiles along the same row of Ag/OTSeo dots (blue curve, shifted vertically for clarity) and a closely located row of silver-free OTSeo crossing points (red curve) as in Figure 5 (main manuscript). The average heights and widths listed on the right refer to all 30 dots and OTSeo crossing points.

The average heights and widths of both the Ag dots and the OTSeo lines obtained from the average contact-mode image [(***trace*** + ***retrace***)/2] in Figure S3 are indeed rather similar to those derived from the semicontact-mode image of the same pattern (Figure 5, main manuscript), thus supporting the view that these images provide valid estimations of the real dot and line dimensions. It further follows from this analysis that the OTSeo height of ~0.4 nm above the OTS background, evident in both the semicontact mode and average contact mode images of the OTSeo lines, appears to be real and may reflect the difference between the vertical extensions of terminal -CH_3 (OTS) and hydrated -COOH (OTSeo) groups [5].

References

1. Hoeppener, S.; Maoz, R.; Sagiv, J. *Nano Lett.* **2003**, *3*, 761–767. doi:10.1021/nl034176l
2. Liu, S.; Maoz, R.; Sagiv, J. *Nano Lett.* **2004**, *4*, 845–851. doi:10.1021/nl049755k
3. Wouters, D.; Willems, R.; Hoeppener, S.; Flipse, C. F. J.; Schubert, U. S. *Adv. Funct. Mater.* **2005**, *15*, 938−944. doi:10.1002/adfm.200400534
4. Zeira, A.; Berson, J.; Feldman, I.; Maoz, R.; Sagiv, J. *Langmuir* **2011**, *27*, 8562–8575. doi:10.1021/la2009946
5. Wen, K.; Maoz, R.; Cohen, H.; Sagiv, J.; Gibaud, A.; Desert, A.; Ocko, B. M. *ACS Nano* **2008**, *2*, 579–599. doi:10.1021/nn800011t
